# Simultaneous Optimization of Acetaldehyde and DMS Concentrations for Better Sensory Quality of Beer Fermented on an Industrial Scale

**DOI:** 10.3390/foods9081043

**Published:** 2020-08-03

**Authors:** Krzysztof Kucharczyk, Krzysztof Żyła, Tadeusz Tuszyński

**Affiliations:** 1 Krakow School of Health Promotion, 31-158 Krakow, Poland; t.tuszynski@kwspz.pl; 2 Department of Food Biotechnology, Food Technology, University of Agriculture in Krakow, 30-149 Krakow, Poland; k.zyla@ur.krakow.pl

**Keywords:** beer production, bottom fermentation, volatile components, large scale, response surface methodology

## Abstract

The levels of selected volatile components that affected the sensory properties of a lager beer were optimized under high-gravity brewing conditions (15.5 °P) in an industrial plant. The influence of different pitching rates (6–10 million cells/mL), aeration levels (8–12 mg/L), times (4.5–13.5 h) of filling CCTs (cylindroconical tanks, 3850 hl), and fermentation temperatures (8.5–11.5 °C) on the contents of acetaldehyde, diacetyl, acetone, 2,3-pentanedion, dimethyl sulfide (DMS), and on the sensory properties of beer were investigated. Response surface methodology (RSM, Box–Behnken design) was used to research the possibilities for optimizing the concentration of selected volatile components and sensory properties of bottom-fermented lager beers. Statistical analyses of the results showed that the experimental factors had a significant influence (R-squared for the original model with no significant lack-of-fit) on some of the volatile components. Based on the Multiple Response Optimization analysis, the values of independent factors that ensured the highest beer sensory quality were the following: a pitching rate of 10 million cells per mL; a fermentation temperature of 11.5 °C; an aeration level of 12 mg/L; and a CCT filling time of 4.5 h. These results proved that RSM modelling can be successfully applied to optimize fermentation and lagering processes in an industrial plant to manufacture lagers of enhanced sensory quality.

## 1. Introduction

The main processes that generate a broad range of flavor components in beer include malting, mashing, and boiling of wort with hops. In addition, yeast cells also synthesize secondary volatile metabolites along with ethanol and carbon dioxide. Although these secondary substances are mostly produced at very low concentrations, they comprise many classes of compounds that are thought to be responsible for the complex aromas of fermented beverages, including beer, wine, and sake [1].

Meilgaard reviewed a list of around 100 flavor constituents responsible for individual flavor notes and discussed their interactions determining the overall character of a given beer brand [2]. More recently, information provided by Alves and coworkers [3] using headspace solidphase microextraction followed by gas chromatography mass spectrometry (HS-SPME/GC-MS) identified an additional 60 volatile organic metabolites at different steps of the beer production processes that contributed to the sensory quality of a lager beer.

The regulation and control of the synthesis of yeast-derived flavor-active beer compounds such as ethanol, CO_2_, carbonyls (aldehydes/ketones), higher/fusel alcohols, esters, vicinal diketones (VDK) (diacetyl and 2-3-pentanedione), fatty and organic acids, and sulfur compounds have also been described [1]. These flavor compounds are known to be intermediates in the catabolic pathways of wort components (sugars, nitrogenous compounds and sulfur compounds) and precursors to the synthesis of amino acids, proteins, nucleic acids, and lipids required for yeast growth.

Aldehydes belong to a group of carbonyl compounds that are characterized by a high flavor-determining potential that exerts a significant influence on the flavor stability of beer. Vanderhaegen et al. [4] stated that acetaldehyde was one of the first compounds whose concentration increase was observed in an aged beer. The content of acetaldehyde in beer varies from 1 to 20 mg/L depending on many processing factors [1,5,6,7]. Higher concentrations of this metabolite not only induce unpleasant “young” or “green” off-tastes, but also participate with phenolics in the formation of beer haze [8]. Acetaldehyde was also listed among the five key contributors to the aged flavor of lager beer [9].

Several vicinal diketones (VDKs) are present in beer, however diacetyl (2,3-butanedione) and 2-3-pentanedione have a significant influence on beer flavor. Mechanisms behind diacetyl synthesis and degradation are very well-understood pathways of yeast metabolism [10]. It is currently accepted that diacetyl is formed from the spontaneous oxidative decarboxylation of surplus α-acetolactate leaking from the valine biosynthetic pathway to the extracellular environment. Similarly, 2-3-pentanedione is formed from α-acetohydroxybutyrate. It seems obvious, therefore, that in order to control the level of VDKs in beer, the correct concentrations of valine and leucine in wort are crucial [11]. At the end of the main fermentation and maturation phase, diacetyl is re-assimilated and reduced by yeast to acetoin and 2,3-butanediol, compounds with definitely higher flavor thresholds.

Unpleasant sulfury flavors whose concentrations must be minimized in the final product originate mainly from malt and hops, but dimethyl sulfide (DMS), the main sulfur volatile, may also be generated from dimethyl sulfoxide (DMSO) by yeast during fermentation or may be the metabolite of wort-spoilage bacteria [11,12]. Dimethyl sulfide (DMS) has a considerable impact on the aroma of beer and may lead to undesirable flavor impressions. The behavior of DMSO in the brewing process has not been investigated in detail. During wort heating in hermetically closed systems 15% of the accumulated DMS was oxidized to DMSO. During fermentation significant DMS formation was observed. DMSO reduction was higher in top fermenting *Saccharomyces cerevisiae* yeast (TUM 19 - Technische Universitat Munchen) than in *Saccharomyces pastorianus* lager yeast (TUM 34/70) [13]. A DMSO reductase encoding the *MXR1* gene has been discovered in *Saccharomyces cerevisiae*, providing a potentially novel tool for controlling DMS levels during fermentation [14]. The dialkyl sulfides, of which DMS is the most common component of beer, have a flavor similar to that of cooked cabbage or sweet corn [15]. The sources of DMS in beer and their relative participation in total DMS concentrations that are produced under various brewing conditions were reviewed in Anness and Bamforth [16]. The authors claimed that DMS is beneficial to the taste and aroma of lager at concentrations below 100 μg/L, but ales usually contain concentrations of this compound that is lower than its flavor threshold (30 μg/L).

Although chemical analysis has contributed much to our knowledge of beer flavor, the sensory characteristics remain a key parameter of beer quality. Knowledge of the flavor chemistry of beer, and particularly of straightforward relationships between beer chemical composition and its sensory properties, remains scarce [15].

Response surface methodology (RSM) offers many advantages over traditional full factorial designs, such as a reduced number of trials necessary for process optimization and more precise results. RSM generates a set of polynomial equations that relate chosen independent (explanatory) variables with response variables. The equations are further explored for prediction of the values of the response variables and for process optimization. This methodology has already been successfully applied to study the effectiveness of commercial enzymes in buckwheat malt mashing [17] and to optimize the characteristic flavor substances in top-fermented wheat beers in pilot-scale experiments [18].

In our previous investigations [6,19,20], the effects of pitching rate, wort filling time, and wort aeration on fermentation, maturation, and volatile compound production in beer produced on an industrial scale were determined in single factor experiments. The purpose of the current work was therefore simultaneous optimization of these parameters by applying the RSM methodology. The optimization was aimed at prediction of relationships among levels of fermentation parameters in an industrial brewery to achieve the most appropriate concentration of volatile carbonyls and sulfur compounds, and to ensure the high sensory quality of a bottom-fermented lager beer.

## 2. Materials and Methods

### 2.1. Experimental Set-Up

The process of beer fermentation and maturation was investigated in industrial cylindroconical fermentation vessels (CCTs). Each fermentation tank was filled with three brews. The final wort volume in each CCT was 3090 hL, whereas the gross CCT capacity was 3850 hL. HGB worts (High Gravity Brew 15.5 °P) were produced using a mix of two pilsner malts from the same two malt houses and with maintaining the constant percentage share of malts from both suppliers. The process of infusion mashing took place at a standard scale temperature of 60–76 °C. Afterwards, the mash was transferred to a lauter tun. After boiling, the wort was cooled to 9 °C and then aerated. Worts were aerated with compressed sterile air under 4 bars of pressure pushed through the wort line with a backpressure of 2.5 bars, during transfer of wort to each of the CCTs and with various intensities so as to have 8–12 mg O_2_ per L of the wort. The concentrations of dissolved oxygen were measured in the pitching wort and after filling of the CCTs using an optical oxygen meter (Mettler Toledo, Columbus, OH, USA) Primary fermentation was performed at 8.5, 10, and 11.5 °C whereas the temperature of the final phase of fermentation was 13 °C. The speed of fermentation expressed as an extract drop was kept as 1.6 °P ± 0.2/day in the first 5 days of the fermentation process. The reduction of an apparent extract content to 3.4 °P marked the end of fermentation and the beginning of maturation process. Both fermentation and maturation were performed in the same tank. High viability of yeast cells and relatively slow fermentation required for the particular brand of beer tested in the study required low standard pitching rate (8 mln cells in 1 mL). Viability of pitching yeasts population was in the range of 95.8% to 99% for each of the 54 runs. Pitching rate was calculated based on the total viable cell number. Depending on the combination of the parameters tested the increase in yeast biomass ranged from 2.2- to 5-fold. Sample collection started after filling the CCTs and was continued during the consecutive 18 days of the production cycle. Sampling from a tank was performed using a sampling device equipped with an installed small pump working in a closed loop system, which let the pump take samples of fermenting wort and mature beer. Samples of beer were taken from CCTs at a point located above the conical portion, 5 m from the bottom of the tank. To obtain representative samples, the circulation pump was running during the whole process, except for the switch off (approximately 24 h) before the yeast harvest. The third generation (yeasts used twice before) of *Saccharomyces pastorianus* brewers’ yeast was used for pitching. The yeast was added to the first of three brews to each CCT. Yeasts were pitched using ABER Instruments Ltd. (Aberystwyth, UK) for rate control, which determined the total viable cell count.

### 2.2. Analytical Procedures

Extract marking was performed using an automatic wort and beer analyzer (Beer Analyzer DMA 4500+ Anton Paar, Graz, Austria), at 20 °C, and the specific weight was measured using an oscillating densitometer. The Tabarié formula was the basis for ‘Alcolyzer’ beer calculations [21].

Qualitative and quantitative analysis of volatile components (the identification was done on the basis of retention time) was performed using gas chromatograph (GC) 8000 (Fisons Instruments, Ipswich, UK) fitted with a flame ionization detector GC-FID (Gas Chromatography—Flame Ionization Detector) and detector GC-ECD (Gas Chromatography—Electron Capture Detector) for detection of diacetyl, 2,3-pentanedione. A mixture of 3-panthenol and n-butanol was used as an internal standard for the determination acetaldehyde and DMS. Concentrations were calculated using a quantitative computer program based on the calculated peak area. Before and after each series of measurements, a comparative analysis was carried out with a beer sample used as a control (reference) batch. The full details of the gas chromatography were given in details in our previous work [22].

### 2.3. Sensory Analysis

Sensory evaluation of bottling beer used a comparison test, with the test sample compared to the reference beer profile. Profile tests involved the evaluation of attributes of the beer, including fruity aroma esters, hops, bitterness, sulfur compounds, sweetness, acidity, fullness, balance, and flavor. The sensory analysis panel consisted of nine employees from the production, analysis, and technology departments. The sample coding procedures used ensured objective evaluations. The sensory quality of beer was evaluated using a gradation scale from 50 to 75 points. The full details of the sensory evaluation of bottled final beer was given in detail in our previous work [22].

### 2.4. Statistical Analyses

Processing factors were tested using the Experimental Design Module of the Statgraphics Centurion XVII ver. 17.1.12 (Professional Edition statistical software, Statpoint Technologies, Inc., Warrenton, VA, USA).

#### 2.4.1. Optimization of the Volatiles and Sensory Quality of Beer

Processing factors that influenced acetaldehyde, diacetyl, acetone, 2-3-pentanedione, DMS concentrations, and the sensory properties of beer were tested using the Experimental Design Module of the Statgraphics Centurion XVII ver. 17.1.12 (Professional Edition statistical software, Statpoint Technologies, Inc., Warrenton, Virginia). The design employed was a fully randomized Box–Behnken design with four factors at three levels each, including pitching rate at 6, 8, and 10 million yeast cells per ml, aeration level of the cold wort at 8, 10, and 12 mg/L, filling time of the CCTs at 4.5, 9, and 13.5 h and fermentation temperature at 8.5, 10, and 11.5 °C, (where the final wort volume in each CCTs was in average 3090 hL) and two blocks, including 3 centerpoints per block, which in 54 runs provided 38 error degrees of freedom. Independent variables, their codes, and actual values are presented in Table 1, whereas the levels of acetaldehyde, diacetyl, acetone, 2-3-pentanedione, DMS concentrations, and sensory analyses were collected in each of the 54 runs. To evaluate the statistical significance of the secondary-order polynomial model, the coefficient of determination (R^2^), and the probability of the lack-of-fit values were calculated. The fitted polynomial equation was then utilized in the “Optimize Response” procedure or analyzed in the form of contour and surface plots to illustrate the relationship between the responses and the experimental levels of the independent variables utilized in this study. The module “Multiple Response Optimization” was employed to study areas where the significant process parameters optimized volatiles and the sensory characteristic of beer. The relationship between the measured exposures and fermentation process parameters were expressed using second-order polynomial equations:

y = β_0_ + β_1_x_1_ + β_2_x_2_ + β_3_x_3_+ β_4_x_4_+ β_11_x_1_^2^ + β_22_x_2_^2^+ β_33_x_3_^2^ + β_44_x_4_^2^ + β_12_x_1_x_2_+ β_13_x_1_x_3_+ β_23_x_2_x_3_ + β_14_x_1_x_4_ + β_24_x_2_x_4_ + β_34_x_3_x_4_ +γ

where y is a volatile concentration or sensory quality; x_1_ is the pitching rate (mln yeast cells/mL of wort); x_2_ is the fermentation temperature (°C); x_3_ is the aeration level (mg O_2_/L); x_4_ is the total time used for CTT filling (h); β_0_ is the intercept coefficient; β_1–4_ are the linear coefficients; β_11_, β_22_, β_33_ and β_44_ are the quadratic coefficients; and β_12_, β_13_, β_23_, β_14_, β_24_, and β_34_ are the interaction coefficients whereas γ is the block effect.

The established models were subjected to ANOVA and Pareto chart (data not shown) analyses, and the non-significant (*p* > 0.05) components were removed from the models. To evaluate the statistical significance of the secondary-order polynomial model, the coefficient of determination (R^2^), and the probability of the lack-of-fit values were calculated.

#### 2.4.2. Multiple Response Optimization Procedures

The module Multiple Response Analysis of the Statgraphics Centurion XVII ver. 17.1.12 (Professional Edition statistical software, Statpoint Technologies, Inc., Warrenton, Virginia), was used to establish the values of technological parameters that simultaneously optimized the content of a few measured responses.

## 3. Results and Discussion

### 3.1. Model Fitting

Within the studied ranges of pitching rate, aeration level, times of filling CCTs, and fermentation temperature, the experimental factors had a significant influence on the acetaldehyde and DMS content, as well as on the sensory quality of the beer, but failed to be significant for diacetyl, acetone, and 2-3-pentanedione concentrations (Table 2).

With the exception of acetone and 2-3-pentanedione concentrations, the temperature of fermentation exerted significant effects on each of the dependent variables, whereas wort aeration affected the DMS levels and sensory characteristics of the beer. It seems to be of interest that the pitching rate and the total time of CCTs filling influenced beer volatile components and the sensory properties of the beer in quite a similar manner. Low R^2^ values for diacetyl, acetone, and 2-3-pentanedione concentrations excluded these models from further consideration.

### 3.2. Polynomial Equations

#### 3.2.1. Acetaldehyde

Table 3 shows the analysis of variance for acetaldehyde content in mature beer after removing insignificant components from the model. The relationship between the four factors and the predicted responses of acetaldehyde concentrations was calculated to be:

y_1_ = 28.93 − 0.317 x_1_ – 0.789 x_2_ − 1.029 x_3_ − 1.405 x_4_ + 0.104 x_3_ x_4_ (1)

where y_1_ denotes acetaldehyde concentration in mg/L, x_1_ stands for pitching rate in mln cells in mL, x_2_ represents fermentation temperature in °C, x_3_ symbolizes aeration level in mg/L, and x_4_ denotes total time of CCT filling in hours.

Within the studied range of the fermentation process parameters, no quadratic component of the model was found to be significant, and the polynomial equation was linear in nature. The subsequent analysis, by means of the Optimize Response module, revealed that over the studied range of independent factors, x_1_ = 10, x_2_ = 11.3, x_3_ = 8, and x_4_ = 13.5 minimized the predicted acetaldehyde to a value of 0.908 mg/L, which is well below the maximal recommended level of this flavor component in beer. There have been multiple attempts to assess the impact of technological factors on the acetaldehyde concentration in the course of beer fermentation. All of the independent factors researched in this study are known to have a direct impact on yeast growth and metabolism and, consequently, on acetaldehyde accumulation in beer. Jonkova and Petkova [5] reported that higher fermentation temperature stimulated early acetaldehyde formation but also increased its reduction rate at later fermentation and maturation phases, leading to its lower concentration in the final beer. Furthermore, in their study, the increased pitching rate significantly reduced the acetaldehyde content in beer. In the previous works performed under conditions almost identical to those employed in this study, we reported quite similar findings [6,20]. However, in another experiment [19], the wort filling time had a significant, positive impact on the course of fermentation and the reduction in acetaldehyde concentrations. Yokoyama and Ingledew [23] also confirmed that CCTs filled multiple times provided better beer quality. Increased breaks in the completion of the filling of CCTs caused better conditions for yeast multiplication at the beginning of fermentation process, particularly because the whole yeast dose was added to the first brew, and prolonged breaks in filling-up the CCTs contributed to an improved vitality of yeast cells in the fermented wort and, consequently, to the reduction in acetaldehyde content. In addition to the total filling time of CCTs, fermentation temperature also had a significant impact on the acetaldehyde production in beer. With increasing temperature, the amounts of acetaldehyde in beer significantly decreased. Similar results were reported by Jones et al. [24], who confirmed that the content of acetaldehyde fell with the increase in the temperature of fermentation to 22 °C. It is known that a reduction in acetaldehyde increases within a temperature range from 5 to 20 °C. It seems obvious that fermentation temperature (x_2_), wort aeration rate (x_3_), and total time of CCTs filling (x_4_) directly influenced the level of oxygen dissolved in the fermenting wort and directly modulated ergosterol, unsaturated fatty acids, bilayer, and mitochondrial structures of growing yeast cells and their potential for acetaldehyde biosynthesis and bioconversion [1,25]. Although early acetaldehyde production rates in *Saccharomyces pasterianus* were found to be strain-dependent, the fermentation lag-phase duration was strongly affected by the pitching rate (x_1_). To the best of our knowledge, Equation 1, for the first time, provides an insight into the relative significance and mutual interrelationships between the basic technological parameters of high-gravity brewing under industrial conditions that may lead to the control and optimization of acetaldehyde content in a lager beer.

#### 3.2.2. DMS

In comparison with acetaldehyde, the corrected model for DMS concentration comprised more significant elements but also lacked any significant quadratic components (Table 4).

Fermentation temperature and pitching rate were among factors that most influenced DMS concentrations in beer, but aeration levels and the filling time of CCTs were also significant, similarly as different two-factor interactions. The nature and power of these effects are given in the following equation:

y_2_ = 79.06 + 16.628 x_1_ + 8.551 x_2_ − 13.067 x_3_ − 9.599 x_4_ − 2.79 x_1_ x_2_ + 1.421 x_1_ x_3_ + 1.063 x_2_ x_4_ (2)

where y_2_ denotes DMS concentration in μg per litre of beer, x_1_ stands for pitching rate in mln cells in 1 mL, x_2_ represents fermentation temperature in °C, x_3_ symbolizes aeration level in mg/L, and x_4_ denotes total time of CCT filling in hours. Using the Equation (2) in the Optimizing Response module, the levels of fermentation parameters that minimized DMS to 42 µg/L were the following: x_1_ = 6, x_2_ = 11.5, x_3_ = 12, and x_4_ = 4.5 (see Table 4). The smallest content of DMS was analyzed in beer when the pitching rate and wort filling times were kept at low levels, whereas the wort aeration rate and fermentation temperature were maximized. From among independent factors, Equation (2) distinctively indicates the predominant role of the pitching rate in affecting levels of DMS in lager beer. The results presented by Verbelen et al. [26,27] did not suggest such a relationship. Different ranges of the inoculum studied by these researchers in laboratory (not industrial-scale) experiments may provide an explanation for these discrepancies. Judging by the significance of the impact on DMS concentrations in beer, the second factor was aeration level. The organo-sulfur volatile concentrations in lager beer were reduced with an increased aeration rate, most likely due to increased purging effects of the CO_2_ formed [14]. As shown in the study by the Verbelen team [28], the exchange of air with pure oxygen reduced DMS content in a lager beer; therefore, it may be concluded that the concentration of oxygen dissolved in fermenting wort may be important for the control of DMS, likely because of more vigorous fermentation [26].

### 3.3. Sensory Analysis

The proper and unchanging sensory quality of beer is one of the most important problems in brewing. The flavor stability and repeatability of the sensory properties of beer was maintained throughout the current study and the marks ranging from 65.7 to 66.7 points ensured “good” overall sensory quality. This formed a background for the assumption that modeling and optimizing process parameters should not exert a negative impact on the sensory quality of the final product. Details of the statistical analyses of sensory data, and particularly analysis of variance of the empirical model predicting the sensory quality of beer have been given in our previous work [22]. It should be emphasized that a higher beer quality resulting from higher fermentation temperatures reported by Engan [29] and Brown and Hammond [30] was claimed to be due to beneficial changes in the volatile components content. The direct relationship between correct wort oxygenation during fermentation and the sensory quality of beer observed in this study is supported by a few previous contributions [28].

### 3.4. Multiple Response Optimization Procedures

The Multiple Response Optimization procedure is a part of the Design of Experiments module of the statistical software used in this work that allows the simultaneous optimization of many dependent factors to be performed. This approach was used to study the possibilities of finding levels where the content of both significant volatiles, i.e., acetaldehyde and DMS, were optimized. Furthermore, we attempted to perform simultaneous minimization of these volatiles and maximization of the sensory quality of beer (“optimize all”). Table 5 compares the results of a single response optimization (which were described earlier) with those originating from the Multiple Response Optimization procedures. This table also includes the predicted values of acetaldehyde and DMS concentrations, as well as the sensory quality of the beer. Findings presented in Table 5 may have interesting theoretical and practical consequences.

In all optimizations, the best temperature of wort fermentation was found to be the highest level of 11.5 °C (+1). With the exception of acetaldehyde optimization, the same was true for the wort aeration level. The best values of the pitching rate and total filling time of CCTs were either at the low (−1) or high (+1) levels, which again suggests the existence of more than one local optimal area. This hypothesis may further be supported by the total filling time, which, at 13.5 h (+1), was optimal for acetaldehyde and sensory properties, but for “volatalites” and “all” optimizations, 4.5 h (−1) was calculated. Perhaps the most important observation that can be made here is the striking similarity of technological parameters that minimized volatiles with those that optimized volatiles along with the sensory quality of beer “Optimize All”. This may be an indication that acetaldehyde and DMS content were among the most important volatiles that affected the sensory quality of the lager beer studied in this work. Although the sensory scores were certainly related to the brewery taste panel training, the tasters, after identifying a particular defect in beer, were also obliged to relate it to specific compounds like DMS, or/and H_2_S, or/and SO_2_. The importance of acetaldehyde as a key contributor to the aged flavor of a lager beer has been established by Saison et al. [9]. In the fundamental work on the taste and flavor of beer, Yonezawa and Fushiki [7] concluded that “Through well-designed experiments coupled with well-trained sensory observers, more precise and more precious knowledge will be compiled without doubt in the future”. In the work presented here, we tested a novel approach in an attempt to find cause-and-effect relationships of sensory evaluation with beer components as suggested by Bamforth et al. [31]. The endeavors reported here were undertaken under standard industrial conditions using the full-scale fermentation plant of a commercial brewery.

## 4. Conclusions

Response surface methodology (RSM; Box–Behnken design) was used to study the relationships between volatile carbonyls and sulfur compounds of a commercial lager beer and its sensory quality. Optimization tests were conducted using four variables: yeast pitching rate; temperature of fermentation; wort aeration level; and total filling time of CCTs. Second-order polynomial equations were employed to find technological parameters that optimize the biosynthesis of volatiles, carbonyls and sulfur compounds, particularly acetaldehyde and DMS content, as well as the sensory quality of beer. Multiple response optimization procedures allowed simultaneous optimization of beer volatiles and the sensory properties to be performed. The levels of independent factors that minimized acetaldehyde and DMS contents (pitching rate 10 million cells in mL; temperature of fermentation 11.5 °C; wort aeration level 12 mg/L; time of filling CCTs 4.5 h) did not change when the sensory quality of beer was also included in the optimization procedure. The results suggest that RSM modelling can be successfully used under industrial conditions for both prediction and control of important fermentation parameters to achieve desirable flavor and aroma of bottom-fermented lager beers.

## Figures and Tables

**Table 1:** Coded and actual values of the variables for the Box–Behenken design.

Independent Variables	Units	Symbol	Coded Levels		
			−1	0	+1
Pitching rate	Mln cells/mL	x_1_	6	8	10
Fermentation temperature	°C	x_2_	8.5	10	11.5
Aeration level	mg/L	x_3_	8	10	12
Total time of CCT filling	h	x_4_	4.5	9	13.5

**Table 2:** Analysis of variance of the selected volatile components and sensory analysis: significance of model components and assessment of adequacy of the models.

Dependent Parameter	Analysis of Variance							
	R^2^	Lack-of- Fit	x_1_	x_2_	x_3_	x_4_	Significant Components of the Model	
		Probability						
acetaldehyde	84	0.6945	0.0241	0.0027	ns	0.0008	0.0389	x_3_x_4_
diacetyl	35	0.4778	ns	0.0284	ns	ns	ns	
acetone	45	0.8257	ns	ns	ns	ns	ns	
2,3-pentanedione	45	0.5567	ns	ns	ns	ns	0.0199	blocks
DMS	76	0.4652	0.0057	0.0045	0.0363	0.0132	0.0115	x_1_x_2_
							0.0397	x_1_x_3_
							0.0192	x_2_x_4_
sensory analysis	71	0.0716	0.0145	0.0010	0.0048	0.0316	0.0014	x_1_^2^
							0.0294	x_1_x_2_
							0.0294	x_1_x_4_
							0.0036	x_2_^2^
							0.0018	x_2_x_3_
							0.0058	x_3_^2^
							0.0164	x_4_^2^

* ns—not significant.

**Table 3:** Analysis of variance: the empirical model for predicting acetaldehyde.

Source	Sum of Squares	Df	Mean Square	F-Ratio	*p*-Value
x_1_	9.6203	1	9.6203	12.49	0.0241
x_2_	33.630	1	33.630	43.68	0.0027
x_3_	0.7902	1	0.7902	1.03	0.3683
x_4_	63.798	1	63.798	82.85	0.0008
x_3_ x_4_	7.0500	1	7.0500	9.16	0.0389
Blocks	0.8676	1	0.8676	1.13	0.3483
Lack-of-fit	34.349	43	0.7988	1.04	0.5630
Pure error	3.0800	4	0.7700		
Total (correlation)	153.18	53			

**Table 4:** Analysis of variance: the empirical model for predicting dimethyl sulfide (DMS).

Source	Sum of Squares	Df	Mean Square	F-Ratio	*p*-Value
x_1_	833.553	1	833.553	29.11	0.0057
x_2_	953.568	1	953.568	33.30	0.0045
x_3_	27.727	1	274.727	9.59	0.0363
x_4_	515.968	1	515.968	18.02	0.0132
x_1_ x_2_	560.455	1	560.455	19.57	0.0115
x_1_ x_3_	258.781	1	258.781	9.04	0.0397
x_2_ x_4_	411.845	1	411.845	14.38	0.0192
Blocks	65.2081	1	65.2081	2.28	0.2058
Lack-of-fit	1619.13	41	39.4911	1.38	0.4201
Pure error	114.53	4	28.6325		
Total (correlation)	5607.77	53			

**Table 5:** Values of independent factors that optimized acetaldehyde, and DMS concentration in the tested beer, its sensory quality, the results from the multiple response optimization (volatiles = acetaldehyde + DMS) and all = volatiles + sensory quality and corresponding predicted values of optimization.

Technological Parameters			Optimum/Goal				
	Levels		Acetaldehyde	DMS	Sensory	Volatiles	All
	−1	+1	Minimize	Minimize	Maximize	Minimize	Optimize
Pitching rate [mln cells/mL]	6.0	10.0	10.0	6.0	6.0	9.9	10.0
Temperature of fermentation [°C]	8.5	11.5	11.3	11.5	11.4	11.5	11.5
Wort aeration level [mg/L]	8.0	12.0	8.0	12.0	10.6	12.0	12.0
Total filling time CCTs [h]	4.5	13.5	13.5	4.5	13.1	4.5	4.50
**Volatiles/sensory Predicted values**							
Acetaldehyde (mg/L)			0.908			3.65	3.69
DMS (μg/L)				42.0		48.4	49.3
Sensory quality (pts)					67.0		66.7

## References

[1] Olaniran A.O., Hiralal L., Mokoena M.P., Pillay B. (2017). Flavour-active volatile compounds in beer: Production, regulation and control. J. Inst. Brew..

[2] Meilgaard M. (1991). The flavor of beer. Tech. Q. Master Brew Assoc. Am..

[3] Alves V., Gonçalves J., Figueira J.A., Ornelas L.P., Branco R.N., Câmara J.S., Pereira J.A.M. (2020). Beer volatile fingerprinting at different brewing steps. Food Chem..

[4] Vanderhaegen B., Necen H., Verachtert H., Derdelinckx G. (2007). The chemistry of beer aging—A critical review. Food Chem..

[5] Jonkova G., Petkova N. (2011). Effects of some technological factors on the content of acetaldehyde in beer. J. Univ. Chem. Technol. Metall..

[6] Kucharczyk K., Tuszyński T. (2015). The effect of pitching rate on fermentation, maturation and flavor compounds of beer produced on an industrial scale. J. Inst. Brew..

[7] Yonezawa T., Fushiki T. (2002). Testing for taste and flavour of beer. Analysis of Taste and Aroma.

[8] Delcour J., Dondeyne P. (1982). The reactions between polyphenols and aldehydes and the influence of acetaldehyde on haze formation in beer. J. Inst. Brew..

[9] Saison D., De Schutter D.P., Overlaet-Michiels W., Delvaux F., Delvaux F.R. (2009). Effect of fermentation conditions on staling indicators in beer. J. Am. Soc. Brew. Chem..

[10] Garcia A.I., Garcia L., Diaz M. (1994). Modelling of diacetyl production during beer fermentation. J. Inst. Brew..

[11] Lodolo E., Kocks J., Axcell B., Brooks M. (2008). The yeast Saccharomyces cerevisiae the main character in beer brewing. FEMS Yeast Resear..

[12] Anderson R., Howard G. (1974). The origin and occurrence of volatile sulphur compounds in british ales and lagers. J. Inst. Brew..

[13] Baldus M., Biermann M., Kreuschner P., Hutzler M., Methner F. (2018). On the behaviour of dimethyl sulfoxide in the brewing process and its role as dimethyl sulphide precursor in beer. BrewingScience.

[14] Hansen J., Bruun S., Bech L., Gjermansen C. (2002). The level of MXR1 gene expression in brewing yeast during beer fermentation is a major determinant for the concentration of dimethyl sulfide in beer. FEMS Yeast Resear..

[15] Jackson J.F., Linskens H.F. (2002). Analysis of Taste and Aroma.

[16] Anness B., Bamforth C. (1982). Dimethyl Sulphide—A review. J. Inst. Brew..

[17] Nic Phiaraise B., Mauch A., Schehl B., Zarnkow M., Gastl M., Herrmann M., Zannini E., Arendt E. (2010). Processing of a top fermented beer brewed from 100% buckwheat malt with sensory and analytical characterisation. J. Inst. Brew..

[18] Cui Y., Wang J., Zhang Z., Speers A. (2015). Enhancing the levels of 4-vinylguaiacol and 4-vinylphenol in pilot-scale top-fermented wheat beers by response surface methodology. J. Inst. Brew..

[19] Kucharczyk K., Tuszyński T. (2016). Effect of wort filling time on fermentation, maturation and acetaldehyde content in beer. CJFS.

[20] Kucharczyk K., Tuszyński T. (2017). The effect of wort aeration on fermentation, maturation and volatile components of beer produced on an industrial scale. J. Inst. Brew..

[21] Miedaner H. (2002). Brautechnische Analysenmethoden: Methodensammlung der Mitteleuropaschen Brautechnischen Analysenkommision. Band II.

[22] Kucharczyk K., Żyła K., Tuszyński T. (2020). Volatile esters and fusel alcohol concentrations in beer optimized by modulation of main fermentation parameters in an industrial plant. Processes.

[23] Yokoyama A., Ingledew W. (1997). The effect of filling procedures on multi-fill fermentations. Tech. Q. Master Brew. Assoc. Am..

[24] Jones H., Margaritis A., Stewart R. (2007). The combined effect of oxygen supply strategy, inoculum size and temperature profile on Very-High-Gravity beer fermentations by Saccharomyces cerevisiae. J. Inst. Brew..

[25] O’Connor-Cox E., Lodolo E., Axcell B. (1996). Mitochondrial relevance to yeast fermentative performance: A review. J. Inst. Brew..

[26] Verbelen P., Van Mulders S., Saison D., Van Laere S., Delvaux F., Delvaux F.R. (2008). Characteristics of high cell density fermentations with different lager yeast strains. J. Inst. Brew..

[27] Verbelen P., Dekoninck T., Saerens S., Van Mulders S., Thevelein M., Delvaux F. (2009). Impact of pitching rate on yeast fermentation performance and beer flavour. Appl. Microbiol. Biotechnol..

[28] Verbelen P., Saerens S., Mulders S., Delvaux F.R., Delvaux R. (2009). The role of oxygen in yeast metabolism during high cell density brewery fermentations. Appl. Microbiol. Biotechnol..

[29] Engan S. (1972). Organoleptic threshold values of some alcohols and esters in beer. J. Inst. Brew..

[30] Brown A., Hammond J. (2003). Flavour control in small-scale beer fermentations. Food Bioprod. Process..

[31] Bamforth C.W., Stewart G.G., Russell I. (2009). Beer: A Quality Perspective.

